# Chicken Authentication and Discrimination via Live Weight, Body Size, Carcass Traits, and Breast Muscle Fat Content Clustering as Affected by Breed and Sex Varieties in Malaysia

**DOI:** 10.3390/foods10071575

**Published:** 2021-07-07

**Authors:** Sara Nematbakhsh, Jinap Selamat, Lokman Hakim Idris, Ahmad Faizal Abdull Razis

**Affiliations:** 1 Laboratory of Food Safety and Food Integrity, Institute of Tropical Agriculture and Food Security, Universiti Putra Malaysia (UPM), Serdang 43400, Selangor, Malaysia; saranematbakhsh@gmail.com (S.N.); jinap@upm.edu.my (J.S.); 2 Department of Food Science, Faculty of Food Science and Technology, Universiti Putra Malaysia (UPM), Serdang 43400, Selangor, Malaysia; 3 Department of Veterinary Preclinical Sciences, Faculty of Veterinary Medicine, Universiti Putra Malaysia (UPM), Serdang 43400, Selangor, Malaysia; hakim_idris@upm.edu.my; 4 Natural Medicines and Products Research Laboratory, Institute of Bioscience, Universiti Putra Malaysia (UPM), Serdang 43400, Selangor, Malaysia

**Keywords:** phenotypic characteristics, chicken breed, village chicken, meat quality, food fraud, food authentication

## Abstract

Nowadays, the high demand for village chickens in Malaysia leads to the fraudulent substitution of indigenous chickens with other cheaper counterparts. Discriminating different chicken breeds based on their phenotypic characteristics is one strategy to avoid chicken adulteration. The main objective of this study was to authenticate and group dominant chicken breeds in Malaysia, including commercial chickens (Cobb, Hubbard, DeKalb) and cross-bred village chickens (Ayam Kampung, Akar Putra). The further discrimination of village chickens from underaged colored broilers (UCBs) (Hubbard, Sasso) was performed based on phenotype traits. The results showed that the breed had a significant effect (*p* < 0.05) on phenotypic characteristics, while the sex effect was not significant for some characteristics. In the first phase, the most remarkable discriminating factors were abdominal fat weight, breast muscle weight, chest circumference, shank length, and wingspan. However, in the second phase, notable variations in phenotypic characteristics between village chickens and UCBs were not detected. Principal component analysis (PCA) showed the successful separation of village chickens from high-performance breeds (broiler and colored broiler). Nevertheless, there was overlap among observations for Sasso and village chickens, which approved the possible similarities in their phenotypic characteristics. This study showed clear breed clustering, which leads to the chicken authentication based on their phenotypic characteristics.

## 1. Introduction

Chicken is an important animal model that fills the evolutionary gap between mammals and other vertebrates [1]. Besides, the increasing demand for poultry meat because of their nutritional values makes the poultry industry one of the largest and fastest-growing agriculture-based industries in the world [2]. Malaysia is one of the highest poultry consumers in the world and the poultry industry is an important source of supply meat protein to Malaysians [3]. The yearly consumption of chicken per capita is near 50 kg, which is the third in the world and the first in Asia [4]. Poultry farming has been the major livestock activity in Malaysia, and the industrial poultry sector in Malaysia has been started with broiler and layer production [5]. There are different chicken breeds that have been supplied for the market demand of chicken meat in Malaysia. These chicken breeds are grouped as high-performance breeds (commercial chickens) and low-performance breeds (Red jungle fowl and Malaysian village chicken) [6]. Commercial chickens can be divided into two main groups, broiler chickens (meat-type) and layer chickens (egg-type). Likewise, commercial broiler chicken strains in Malaysia consist of Ross, Cobb, etc., that are products of intense and successful selection programs for rapid growth and body conformation, markedly in terms of breast muscle development [6,7]. Broiler chicken meat is important to every household in Malaysia as it is included in people’s diets at least once a week either at home or at local restaurants [3]. Another commercial chicken in Malaysia is layer chicken, in which five-layer chicken types are available in the market, including Hisex Brown, Lohmann Brown, Dekalb, ISA Brown, and Novogen [8]. Alternatively, Red jungle fowl breeds are a slow-growing type of chickens that are protected by the Malaysian government and have a potential niche market in the future [7].

The present Malaysian village chicken, commonly known as “Ayam Kampung”, is the result of crossbreeding of the Red jungle fowl with mixed exotic domestic breeds brought by Europeans, mainly the British [9]. The original Malayan fowl were the descendants of the southeast Asian jungle fowl (*Gallus bankiva*) through natural mating and selection [5]. Village chicken is popular in many southeast Asian countries such as Malaysia and it has been superior compared to commercial broiler chickens in terms of wholesomeness and health benefits. Recently, rising awareness of people about using drugs such as antibiotics in commercial poultry production led to a significant increase in demand for free-range native chicken, such as village chicken, which covers a large niche market compared to previous years because of emergent food safety and animal welfare concerns [10]. Today, the marketing and export of village chickens to supply the growing market already exist in southeast Asia [11]. Akar Putra is a local Malaysian chicken, and the crossbreeding occurred when the wild jungle fowl entered the Universiti Putra Malaysia farm and mated with the university’s chickens (Ayam Kampung) in the research farm [12].

Moreover, currently, food fraud is a big business and is covering various food commodities, more notably meat and meat products, thus the main concern of food safety regulators and trading partners is the ability to confirm the security and authenticity of food products. In the same way, due to the high demand for village chicken in Malaysia, there is a concern that commercial chicken, due to their cheap cost, are claimed as the village chicken to fraud consumers for economic gain since there is no strategy to authenticate different chicken breeds in Malaysia. According to several studies, the most reliable method for differentiating among different breeds of animals is a genomic study to understand genome differences, and therefore discovery of genomic biomarkers to facilitate discrimination of different chicken breeds is of paramount importance. However, as stated by FAO, the high-efficiency molecular characterization of different animal breeds should be followed by phenotypic characterization [13]. Phenotypic characterization is the process of identifying distinct breed populations and describing their external and production characteristics in different environments and management systems [13,14]. There are different methods to characterize animals, ranging from linear measurement of morphological traits to the use of molecular techniques [15]. For example, phenotypic and morphological measurements have been used to characterize and differentiate various chicken breeds [16,17,18], and different molecular methods including microsatellites and single-nucleotide polymorphisms (SNPs) have been used to investigate the genetic structure of different chicken populations [19,20]. The most common methods for phenotypic characterization are canonical discrimination and multivariate analyses [21]. This study focused on the economically important traits, including body length, wingspan, shank length, chest circumference, live weight, carcass weight, dressing percentage, abdominal fat weight, breast muscle weight, and intramuscular fat percentage. It is demonstrated that intramuscular fat (IMF) had a significant role in the sensory quality of chicken meat by positively affecting the juiciness, flavor, and tenderness of the meat. IMF content in the muscle varies according to anatomical muscle origin, age, breed, genotype, diet, and the rearing conditions of livestock [22].

There have been several studies that investigated the phenotypic characteristics and meat quality of various chicken breeds. For instance, the comparison of breast muscle meat quality in two broiler breeds at their market age, including 120-day-old Beijing-you-(BJY) and 42-day-old Arbor Acres (AA) chickens, revealed that the phospholipid and essential fatty acid content in BJY chickens were higher than those in AA chickens, in which phospholipids contain most of the polyunsaturated fatty acids, such as linolenic acid and arachidonic acid [23]. The meat characterization analysis of three breeds in Malaysia, including Red jungle fowl, Malaysian village chicken, and commercial broiler, showed that collagen content, PH, cooking loss, and shear force values in Red jungle fowl and Malaysian village chicken were significantly higher compared with commercial broiler [7]. The same researchers performed another analysis to determine and compare the carcass composition in the three mentioned chicken breeds, revealing that the broiler chicken had significantly higher mean value for the whole carcass weight, meat weight, bone, and fat weight, followed by Malaysian indigenous chickens and Red jungle fowl [6].

**Figure 1:**
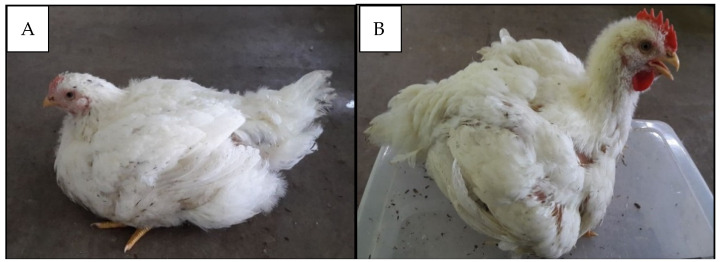

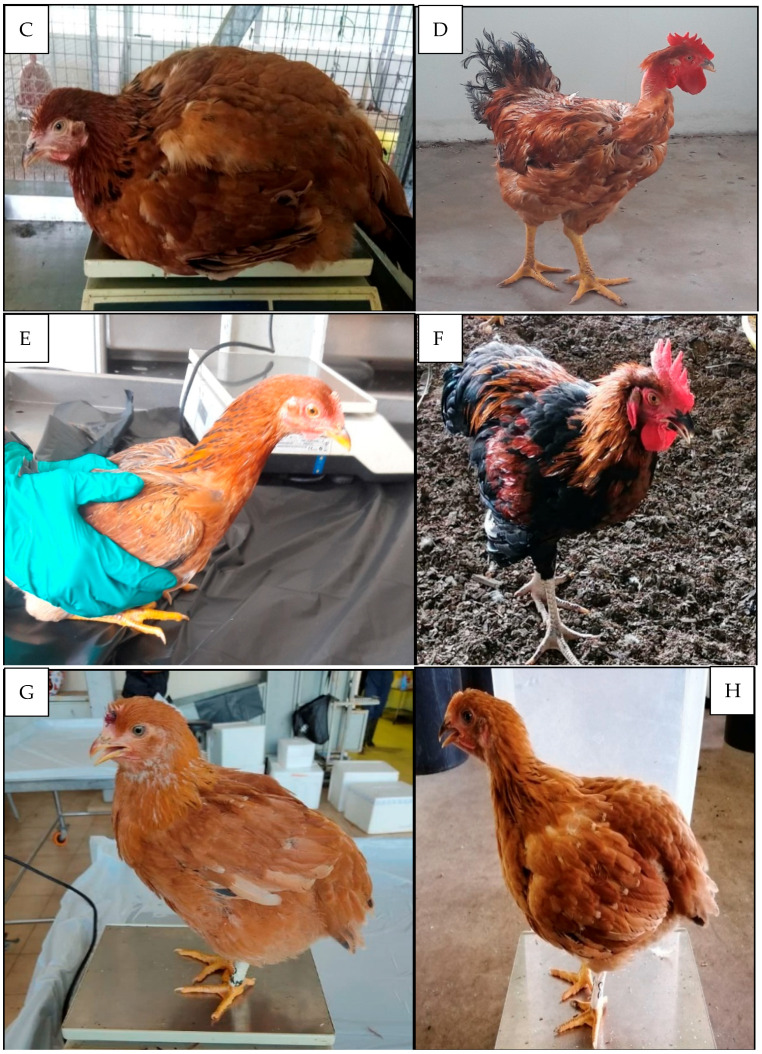

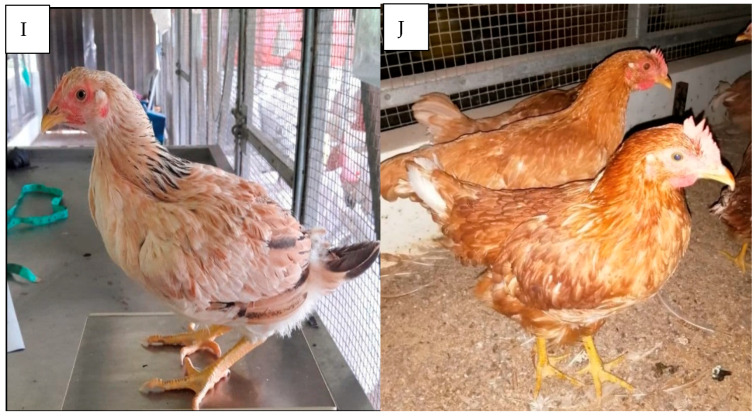
Appearance of 10 chicken types used in this study. (**A**) broiler-Cobb (Female), (**B**) broiler-Cobb (Male), (**C**) colored broiler-Hubbard (Female), (**D**) colored broiler-Hubbard (Male), (**E**) naturally mated village chicken (Ayam Kampung) (Female), (**F**) naturally mated village chicken (Ayam Kampung) (Male), (**G**) underaged colored broiler-Sasso (Female), (**H**) underaged colored broiler-Hubbard (Female), (**I**) selective bred village chicken (Akar Putra) (Female), and (**J**) layer-DeKalb (Female).

The first objective of the present study was authentication and grouping of four chicken breeds, including three commercial stocks, broiler-Cobb, colored broiler-Hubbard, layer-DeKalb, and naturally mated village chicken (Ayam Kampung) at their market age in Malaysia based on their phenotypic characteristics (Figure 1). The second objective of this study was to authenticate and differentiate naturally mated village chicken (Ayam Kampung) (NMVC) and selective mated village chicken (Akar Putra) (APVC) from underaged colored broiler, including Hubbard and Sasso (UCBH, UCBS), based on their growth performance and carcass traits (Figure 1).

To the best of our knowledge, no information has been reported on the village chicken authentication based on phenotypic characteristics before and after slaughter in Malaysia. In this study, each chicken breed was supplied from its natural rearing environment, since it is well-documented that apart from genetic make-up, the environment plays a crucial role in the phenotypic variation of chicken breeds [24,25].

The limitation that needs to be highlighted in this study is that no farm has agreed to supply underaged colored broiler breeds (UCBH, UCBS); therefore, they were reared in the research farm facilities at the Universiti Putra Malaysia (UPM). However, the same starter and grower feed, which were used for chickens in first phase (commercial feed), were provided for underaged colored broiler chickens.

## 2. Materials and Methods

### 2.1. Ethics Statement

All the animal experiments performed in this study were approved by the Institutional Animal Care and Use Committee (IACUC), Universiti Putra Malaysia (UPM), with the approval number: UPM/IACUC/AUP-R022/2019, and the experiments were carried out in accordance with the approved guidelines established by this committee.

### 2.2. Experimental Population and Sampling

#### 2.2.1. First Phase: Commercial Chicken Breeds vs. Village Chicken (Ayam Kampung)

In this study, four breeds of chickens at their market ages, which have been bred as the end products and known to differ significantly in growth performance and other economically important traits, were provided from different farms in Malaysia. The population of chicken breeds including broiler (Cobb), colored broiler (Hubbard JA57), and layer (DeKalb) were purchased at the age of 6, 10, and 48 weeks (not functional for laying eggs) from the local farms, including Ladang Bukit Mertajam, Ladang Permatang Tinggi, and Ladang Butterworth in Penang, respectively. The village chicken (Ayam Kampung) population was provided at the age of 4.5 months from the farm in Ladang Pahang Tua in Pahang.

All chickens investigated in this study are commercial chickens, which are raised to become poultry meat. However, the commercial layers which are raised to produce table eggs [4] can be used as a meat source after not being functional in egg production. In Malaysia, a broiler-housing system usually consists of several broiler sheds, each accommodating 20 to 25,000 chickens, while laying hens are usually raised in cages, and each farm has multiple sheds, which can accommodate between 20,000 to 60,000 chickens in the cages. In these farms, there are automated equipment for feeding and drinking lines, heating, feed storage, ventilation, light, and control system [4]. The traditional housing system for village chicken is free range, however, recently, farmers combine the free range and semi-intensive rearing systems for mass production of village chicken [5].

The field survey design and data collection procedure were performed according to FAO’s phenotypic characterization of animal genetic resources [26], with the help of experts from the Institute of Tropical Agriculture and Food Security (ITAFoS), UPM. The number of chickens for phenotypic measurement consisted of 34 chickens/each breed (17 male and 17 female), with the exception of the layers (17 female). The layer-type males are normally culled immediately after hatch because they have a long fattening period, unfavorable feed efficiency, and non-appealing appearance of the products [27]. All mature chickens from each breed were supplied directly from the farms with normal and standard rearing environment specific to each breed, because almost all traits (phenotypes) can be affected by both genetic and environmental factors (such as temperature, type of feed, etc.). Chickens in both phases were fed on the commercial starter and grower feed (Gold Coin Feedmills (M) Sdn Bhd). Based on the farm information, the feed for the village chicken had a base of commercial gold coin with some extra add-in ingredients, such as corn. The commercial feed ingredient consisted of soybean meal, corn, other grains and grain by-products, animal protein, vegetable oil, salt, calcium carbonate, coccidiostats, and approved antimicrobials. Nutritional values of the commercial feed are presented in Table 1. According to the Malaysian Feedmillers Association (MFA), corn is the main component of the typical chicken feed mix and contributes 55% of the feed, soybean is the main source of protein and makes up 25% of the feed, 5% of the feed is palm oil and rice bran, and the remaining 15% is vitamins, minerals, bone meal, fish meat, wheat bran, wheat pollard, and corn gluten meal [4].

#### 2.2.2. Second Phase: Village Chickens vs. Underaged Colored Broiler Breeds

The farms rearing colored broiler chickens refused to provide underaged (42 days/1 kg) colored broiler breeds (Hubbard and Sasso). Thus, in the second phase of the project, the colored broiler (Hubbard JA57) and colored broiler (Sasso Ruby XL) were reared at the research farm in ITAFoS/UPM from July 2020 to September 2020. The rearing period was from day 1 until they reached the weight ranging from 1 to 1.3 kg to make their phenotype comparable with village chickens at their market weight (1–1.3 kg). Likewise, one-day-old chicks from both breeds, Hubbard JA57 (*n* = 17) and Sasso Ruby XL (*n* = 17), were raised for 6 weeks (42 days) under the floor husbandry conditions in 2 pens with 17 chickens each on a broiler diet (commercial gold coin).

The rearing conditions followed commercial broiler rearing guidelines. Briefly, chicks were raised in a brooder guard with two heating lamps from day 1 to 14 days of age. Feed and water were available ad libitum from 2 drinkers and 4 feeders in each pen. The chicks used water mixed with the anti-stress supplement for 3 days and then normal water was used until day 42. Regarding feed, chicks were fed on the broiler starter (Gold coin) until 21 days of age, then the feed was changed to the broiler grower (Gold coin) until 42 days of age (Table 1). At 7 and 21 days of age, chicks were vaccinated against Marek and Newcastle’s disease. No other veterinary treatment was applied during this study. When chickens reached the targeted weight (1–1.3 kg), they were slaughtered in the slaughterhouse located in the Faculty of Agriculture, UPM.

The data for naturally mated village chicken (Ayam Kampung) was provided from the first phase of the current study. The selective mated village chickens, Akar Putra, were supplied from Azil Green Resources Sdn Bhd, Selangor, at their market age (2.5 months, 1–1.3 kg) (*n* = 17). It should be noted that both village chicken breeds were fed on a commercial feed (Gold coin). The phenotypic characteristics and slaughtering methods and conditions exactly followed the first phase procedure, which are mentioned in the following paragraphs.

### 2.3. Phenotypic Characterization

#### 2.3.1. Live Weight and Body Size Measurement

Before slaughter, chickens (both phases) went through the 12 h feed withdrawal period in the farm at ITAFOS, UPM. Then, all the chickens for each breed were weighed using an electronic weighing scale and the live body weight (g) was recorded. Subsequently, other body size measurements including body length, wingspan, shank length, and chest circumference were recorded in cm using a measuring tape. All the data collection for phenotypic characterization was done based on guidelines provided by FAO, 2012, on phenotypic characterization of animal genetic resources [26]. All the measurements were taken by the same person to avoid between-individual variations.

#### 2.3.2. Carcass Traits’ Measurement

All chickens (both phases) were transferred to the slaughterhouse located in the Faculty of Agriculture, UPM. Briefly, chickens were sacrificed by cutting of the jugular veins, and then birds were left for about 1.5 to 2 min for complete bleeding. After bleeding, chickens were manually de-feathered and de-skinned. All fats found inside the chicken abdominal cavity were removed and weighed as abdominal fat weight (g). The carcass weight (g) was calculated using electronic weighing balance by removing the head, neck, leg, feathers, skin, blood, wing, viscera, abdominal fat, and all inside organs. The dressing percentage was determined by dividing the warm carcass weight by the live weight of the chicken and expressed as a percentage. Subsequently, whole breast muscle with bone was removed from the carcass and individually weighted and recorded (g). The whole breast tissue (pectoralis major and minor) of all chickens after weighing were separated from bones and wrapped in aluminum foil and kept at −80 °C for further analysis.

#### 2.3.3. Intramuscular Fat Content Analysis

The intramuscular fat analysis of breast tissue (pectoral muscle) was performed using the Foss Soxtec Auto Fat Extraction System 2050 based on the official AOAC 991.36 method [28]. First, it should be noted that breast tissue had approximately 70% moisture content, which can interfere with the organic solvent during the fat extraction process. Therefore, 20 g of minced tissue need to be put in the oven (106 °C, 90 min) to lessen its moisture content. In this study, the calculation of crude fat was based on the dry weight of breast tissue (pectoral muscle). Therefore, the moisture content of dry tissue samples was determined and recorded based on the AOAC method [29].

Afterward, 2 g of well-dried sample was weighed to the extraction thimble using an analytical balance (accurate to 0.1 mg). Then, the thimbles were attached to the Soxtec set and placed on top of the extraction cubs filled with 70–90 mL of petroleum ether (40–60 °C) as the extraction solvent. The control unit was used for running the fat extraction process, in which based on the Foss application note 3127 (extraction fat in meat and meat products), extraction times in each stage were, boiling time 25 min, rinsing time 30 min, and recovery time 9–10 min. The extraction temperature based on the sample type and extraction solvent was set at 160 °C.

In the first phase, 20 chickens from each breed were randomly (10 males, 10 females) selected for the intramuscular fat analysis, and in the second phase, a total number of 10 chickens (female) for each breed were used, except for Akar Putra, where 8 female chickens were selected for analysis. The rest of the samples were used for phenotypic characteristics, not pertaining to this part.

##### Calculation of Intramuscular Fat Content

Based on the application note AN3127 [28], the intramuscular fat percentage (IMFP) was calculated using the following formula:
$\%\;\mathrm{Fat}=\frac{W3-W2}{W1-M}\times 100.$W1 = Sample weight (g)W2 = Extraction cub weight (g)W3 = Extraction cub weight (g) + extracted fat weight (g)M = Moisture content weight (g)

### 2.4. Statistical Analysis

Experimental data for intramuscular fat were conducted in three replicates and the results for all phenotype data were presented as mean ± standard deviation. Data were evaluated statistically using MINITAB software Version 17.0 (Manufacturer, Sydney, NSW, Australia). The general linear model (GLM) was used to analyze the significant effect of breed (broiler, colored broiler, layer, and village chickens) and sex (male, female) on different phenotypic characteristics. Furthermore, the Tukey’s pairwise comparison was performed by considering the probability level of *p* < 0.05 as statistically significant to see which breeds are significantly different in terms of their phenotypic characteristics. Moreover, the MINITAB 17.0 software was used to conduct a principal component analysis (PCA) to evaluate the relationship between different chicken breeds and classification of chicken breeds based on their phenotypic characteristics.

## 3. Results and Discussion

### 3.1. First Phase: Breed Effect

It is well-documented that the genetic background of various animal breeds is one of the influential factors, which has a dominant effect on livestock phenotypic characteristics [30,31]. Since even close species/breeds may not share quantitative trait loci because of recombination [32], it can be concluded that each breed has its own genetic makeup, which leads to specific phenotypes. Likewise, the study by Mohsin et al. underlined the importance of goat breed on the chemical composition of milk, in which chemical composition of milk can be used as the potential criteria for the classification of goat breeds [33]. Host genomic variants analysis in pigs demonstrated that the host genome can impact the phenotypic traits by instigating a change in the gut microbiome composition that changes the phenotype (fat deposition) [31]. In chicken, genetic selection, which has been made to improve production traits in broiler chickens and reproductive traits in laying hens, caused remarkable differences in the growth rate and development traits among chicken breeds. These differences can be seen in traits such as growth rate, muscle fat content, feed consumption, and muscle development, which are due to both genetic and epigenetic modifications [34]. Likewise, the quantification of genetic variants related to economically important traits, muscle, and meat quality traits in chicken revealed that there were significant amounts of genetic variation for these traits among and within commercial broilers, layers, and traditional chickens [35].

In this study, evaluating the effect of breed on different phenotypic variables revealed that there were significant differences (*p* < 0.05) among the four dominant breeds available in Malaysia. Therefore, these variations in the phenotypes among four breeds suggested that as expected, genetic background/breed had a significant effect on the phenotypic traits in different chicken lines. The results (average value ± standard deviation) of the chicken breed and sex effects on all recorded phenotypic characteristics before and after slaughter are shown in Table 2. In the first phase, the village chicken population, which was investigated and compared with other chicken breed populations, was naturally mated village chicken (Ayam Kampung).

#### 3.1.1. Live Body Weight and Linear Body Measurements

Results of the breed effects on body size traits (Table 2) indicated that four breeds in this study can completely be divided into two distinct groups, including fast-growing chickens (Cobb and Hubbard) and slow-growing chickens (DeKalb and village chicken), based on the wingspan and chest circumference, of which the latter is the indicator of fleshing in chicken [36]. However, other body size traits, including body length and shank length, were not a significant discriminator of chicken breeds. For instance, Cobb and village chicken as well as Cobb and DeKalb had the same value for the body length and shank length, respectively. While Hubbard had a significantly higher mean value for both traits (Table 2).

Wingspan and chest circumference values in Hubbard and Cobb, as fast-growing chicken breeds, were significantly (*p* < 0.05) higher than slow-growing chicken breeds, including DeKalb and village chicken. Likewise, the higher mean value for both traits belonged to Hubbard, followed by Cobb. In fact, Hubbard and Cobb had a significantly (*p* < 0.05) higher value for most of the phenotypic variables, including wingspan, chest circumference, live weight, carcass weight, and breast muscle weight, compared to village chicken and DeKalb (Table 2).

Moreover, chest circumference in the broiler (Cobb) had a dominant higher value compared to Malaysian village chicken, which is approved by the study of Lokman et al. [6] who revealed that broiler (Ross) had significantly higher chest circumference compared to Malaysian village chicken.

Overall, Hubbard had a higher mean value for body weight and linear body measurements compared to other breeds. Accordingly, Hubbard was heavier and longer/taller than other breeds in this study at their market age (Table 2).

#### 3.1.2. Carcass Characteristics

Carcass traits were strongly influenced by chicken types, and the most significant differences were observed in value for breast muscle weight. Breast muscle weight was the heaviest (*p* < 0.05) in the Cobb population, followed by Hubbard, DeKalb, and village chicken. In other words, breast muscle weight in Cobb had a significantly (*p* < 0.05) higher mean value (489.706 g) compared to other breeds (Table 2), which confirmed the main goal of commercial broiler chicken production for achieving high-yield breast muscles as the most valuable piece of chicken carcass [6]. In contrast, village chicken had the lowest mean value (*p* < 0.05) for breast weight (Table 2), which had conformity with its low-growth rate. The second heaviest breast muscle value belonged to Hubbard (359.974 g), followed by DeKalb. DeKalb is a slow-growing chicken, which had a higher proportion of breast weight (248.824 g) compared to the village chicken at their market age, while the breast muscle in DeKalb was lighter and flatter compared to commercial broiler examined in this study (Table 2). This result is in agreement with the study of Mueller et al., who indicated that the breast weight (g) of commercial fast-growing broiler (Ross PM3) (521 g) was significantly higher than the colored broiler (Sasso 51) (335 g), layer (Lohmann Brown plus) (130 g), and two traditional chicken lines. In which, the breast weight in the layer line and two traditional chicken lines was significantly lower than the commercial broiler and colored broiler lines [27].

Regarding body weight, since four chicken breeds under study had different market ages, it is not surprising that there were significant differences in live and carcass weight among the breeds. However, Cobb and Hubbard significantly grouped together and differentiated from slow-growing chicken breeds (village chicken and DeKalb) based on the live and carcass weight (Table 2). These results are in agreement with the study of Lokman et al. [6] that indicated that the mean whole carcass weight in the high-performance breed (broiler-Ross) was higher compared with a lower-performance breed (Malaysian village chicken). 

Dressing percentage (DP) is an important criterion for the evaluation of the slaughter value of broiler carcass. Broiler chickens normally possess a high DP, accounting for 74.81% compared to other breeds [37]. In this study, Cobb had a significantly (*p* < 0.05) higher DP (71.81%) compared to other breeds. The high DP is an indicator of the highest carcass yield in the Cobb as a genetically improved chicken breed to reach the highest weight in a short time. Furthermore, as mentioned earlier, Cobb had the heaviest breast muscle weight compared to other breeds, which can be matched with its highest DP. Subsequently, the lowest DP was observed in the DeKalb (60.76%) (Table 2). Probably, it can be explained by the observation during slaughter, in which some of the layer chickens still had eggs inside their stomach that caused higher live weight compared to the carcass weight, and consequently, lower DP. Despite the almost same mean live weight value in broiler and colored broiler breeds, DP in Cobb was higher than Hubbard, followed by village chicken and DeKalb (Table 2). This result was consistent with the results of Mueller et al., who showed that commercial fast-grower chickens (Ross PM3) had a significantly higher DP, followed by colored broiler (Sasso 51), traditional chicken breeds, and layer [27].

Since excessive fat accumulation in the abdominal cavity is the main problem of the chicken production industry, specifically broiler production, abdominal fat content is the major phenotypic indices of fat trait [38]. In this study, chicken breeds significantly (*p* < 0.05) influenced abdominal fat weight (AbFW). Likewise, Hubbard had significantly higher AbFW (72.56 g) compared to other breeds, followed by DeKalb (58.51 g), Cobb (29.28 g), and then village chicken (0.34 g) (Table 2). The most notable difference was observed in the AbFW of village chicken, being extensively lower than Hubbard and Cobb. Similarly, another study indicated that the AbFW was in the highest value in the colored broiler (Sasso 15) compared to broiler-Ross, layer, and traditional chickens. They highlighted that the housing system, where chickens move minimally, and the dense diet high in energy were the reasons for an extraordinary great proportion of abdominal fat in colored broilers [27]. The study by Lokman et al. [6] also revealed that broiler (Ross) had the highest mean fat weight compared to Malaysian village chicken, which is in agreement with the present study. In other investigations, the proportion of abdominal fat in commercial broilers was significantly (*p* < 0.05) higher compared with layers [27,35] due to the genetic selection of commercial broiler to produce maximal meat, which increases the fat content [39]. In contrast, in the present study, the layer-DeKalb had significantly higher AbFW compared to the Cobb. The reason for this variation can be explained by the differences in layer breeds, slaughter age, and the maturity level of chickens in the current study compared to other investigations. For instance, since the main concern of this research was to investigate common chicken breeds in the Malaysian market, layer chickens were inspected at their late-laying period (48 weeks), in which their laying ability is extremely reduced [40], and can be used as meat in the market. It is worth noting that there is increased fat deposition and decreased lipid metabolism ability in late-laying hens compared to peak-laying hens, and therefore layer chickens in this study had notable fat content in the abdominal cavity [41].

On the other hand, village chickens had the lowest abdominal fat content, which is consistent with their nature, including eating behavior and maximal activity during the rearing duration, that leads to strong and tough muscle and low AbFW [6]. Similarly, another study in China showed that a local chicken breed named Biacheng-You chickens had much lower abdominal fat weight compared to Arbor Acres broilers as a commercial chicken breed [39]. Comparison of fatness and meat quality traits among Kampung (Indonesia’s native chicken), Arabic (local chicken), and the laying-type chickens showed similar findings in which type of chicken had affected the levels of abdominal fat and fat level of meat. Correspondingly, it is revealed that laying-type chickens had significantly higher abdominal fat content and meat fat (g/100 g) compared to the other two breeds, indicating different metabolism rate or energy retention patterns for fat establishment and development in each chicken breed [42]. Moreover, abdominal fat variation also happened among broiler chickens, where among three broiler breeds (Ross 308, Hubbard Flex, and Hubbard F15), Hubbard Flex had the highest abdominal fat content [16].

Overall, it can be concluded that phenotypic characteristics of chickens can be extensively influenced by chicken breeds. Therefore, phenotypic traits including body size and carcass traits can be a significant indicator for grouping and differentiation of different chicken breeds before and after slaughter at their market age. In this study, the most remarkable differentiating factors can be breast muscle weight, chest circumference, body size, carcass weight, and abdominal fat weight. Furthermore, among all studied breeds, Hubbard had a higher value for all traits except for carcass weight, dressing percentage, and breast weight, whereas Cobb had higher values for these phenotypes. In contrast, village chicken had the lowest value for live and carcass weight, breast weight, and abdominal fat weight. Based on the results, it can be observed that DeKalb held the significant middle stage for most variables. However, the highest and lowest abdominal fat weight and dressing percentage respectively, belonged to the DeKalb.

### 3.2. First Phase: Sex Effect

Regarding the sex effect on phenotypic characteristics (Table 2), three breeds of chicken were considered for analysis (Cobb, Hubbard, and village chicken), since in this study, only female layer chickens were used for investigation. The results indicated that although male chickens (*n* = 51) had the higher mean value for all measurements, except for AbFW, compared to female chickens (*n* = 51), sex had no significant effect (*p* < 0.05) on wingspan, dressing percentage, breast muscle, and abdominal fat weight in all three breeds (Table 2). The results of this study corroborated the previous finding, revealing that the mean value for all traits (body weight, carcass weight, and breast muscle weight) were higher in males than in females, except for AbFW, which showed the greater impact of hormones for fat deposition in females [43]. Another investigation on sex effect on abdominal fat yield showed that female broilers significantly deposited more abdominal fat than male broilers [44].

However, some phenotypic characteristics significantly (*p* < 0.05) differed among males and females, including body length, shank length, chest circumference, and live and carcass weight (Table 2). In which, male chickens had a significantly higher value than female chickens. Therefore, it can be concluded that male chickens were heavier and larger than females in terms of body weight and some body size traits at the same market age, which helped us to phenotypically differentiate chicken based on sex. Present results confirm those of Hussein et al. [37], who found that males had higher significant carcass parameters (live weight, carcass weight, dressing percentage, and breast weight) compared to females between breeder and broiler chickens from Ross 308. Sex effects were also reported by López et al. [45], who revealed that broiler males had higher (*p* < 0.05) carcass weight, live weight, and breast weight than females, but lower dressing percentage [45]. Accordingly, in the current study, there were no significant differences between males and females in terms of their dressing percentage. Moreover, a study by Azahan [46] also revealed that Malaysian male village chickens (Ayam Kampung) had significantly superior body weight compared to female chickens, which was more obvious between weeks 9 and 15 of age. Other similar studies also showed that male chickens were heavier than females in different chicken breeds [17,24,47]. Besides, significant differences in body length, neck length, chest girth, wingspan, and body weight measurements have been indicated between male and female Tanzanian local chicken breeds. The least-square means for the chest girth, wingspan, and shank length were significantly higher in male chickens compared to females in all three local Tanzanian chicken ecotypes [15].

As can be seen in Table 2, the interaction of breed and sex (B × S) had no significant effect on most of the phenotypes; however, body length, shank length, and chest circumference can be significantly (*p* < 0.05) affected by both breed and sex factors. Correspondingly, the interaction between sex and chicken ecotype effects in three local Tanzanian breeds was only significant for the wingspan, shank length, and chest girth [15].

### 3.3. First Phase: Intramuscular Fat Content Analysis

Intramuscular fat (IMF) is the deposited fat between the muscle and is the energy reservoir matter of livestock. Moreover, IMF is an important indicator of chicken meat quality and is significantly correlated with the tenderness, flavor, and succulence of the meat [48,49]. Since genetics is a major factor influencing meat quality of chickens [23], this section aimed to investigate the effect of breed and sex on IMF percentage (IMFP) of breast muscle. The mean value ± standard deviation for IMFP in breast muscle of four chicken breeds is presented in Table 3.

The current investigation revealed that breed had a significant impact on the IMFP in different chicken breeds. Accordingly, chicken breeds were classified into three different groups based on the IMFP, the first group consisted of layer-DeKalb, which had significantly higher IMFP (3.61%) compared to other breeds. Subsequently, the second group comprised of commercial broiler chickens, Cobb, and Hubbard, which significantly differed from village chicken and DeKalb and held the middle stage of IMFP (2.85% and 2.44%, respectively). The last group belonged to the village chicken, which had the significantly lowest IMFP (1.24%) compared to other breeds (Table 3).

The highest IMFP in the DeKalb can be a result of slaughter age, as they were in the later-laying period (48 weeks) and hens have higher fat deposition ability in the late-laying period. Consistent with our results, a study by Zhang et al. revealed that later-laying-period Gushi (Chinese local breed) hens showed a higher proportion of IMF content and serum lipid level compared to young laying hens [41].

Moreover, the IMFP was greater in commercial broilers including cobb, followed by Hubbard compared to village chicken (Table 3). This result is almost similar to other studies which suggested that broiler chickens have more fat content in breast muscle, followed by colored broiler (Sasso) and traditional chicken breeds [27]. The study of Guo-Bin et al. on Chinese native chicken breeds revealed that breed is the key factor that affects IMF content in chicken. They indicated the IMF content of breast muscle in Anka chicken was significantly higher than those of local chicken breeds (Wenchang and Rugao breeds) [48], which is consistent with the current study in which the IMFP of broiler, colored broiler, and layer is significantly higher than the local Malaysian village chicken.

In terms of sex effect (Table 3) on IMFP, layer chickens again were not considered. The results indicated that although female chickens (*n* = 30) had a negligible higher mean value of IMFP compared to male chickens (*n* = 30), sex was not a statistically significant factor affecting IMFP in breast muscle of chicken breeds. Similarly, the interaction of sex and breed also had no significant effect on the IMFP. The results of this study corroborated the previous finding by Chang et al., which revealed that the IMFP of hens was higher than that of cocks in all three breeds under study, however, this difference was only significant in the Wenchang chicken breed population [46]. Similarly, in this study, female chickens had negligible higher IMFP than male, but only in Hubbard were the significant (*p* < 0.05) differences among sex detected (Table 4). As can be seen in Table 4, there were significant differences in IMFP among male chickens in each breed, while in female chickens, Cobb and Hubbard grouped together and significantly separated from female village chicken.

Overall, in this study, the IMFP of breast muscle can be significantly affected by chicken breed. Likewise, IMFP can group commercial broiler, cobb, and Hubbard and separate them from DeKalb and village chicken. This information can be a foundation on improving the meat quality in meat-type chickens available in Malaysia.

### 3.4. Second Phase: Malaysian Village Chickens’ Differentiation from Underaged Colored Broiler Breeds

The Malaysian village chickens, commonly known as Ayam Kampung, are the consequences of crossbreeding of Red jungle fowl and mixed exotic breeds brought by Europeans (mainly British) through natural or selective mating. According to the Department of Veterinary Service Malaysia (DVS), there are significant variations in phenotypic characteristics and production performance of various village chicken breeds available in Malaysia since the breeding and mating system was unplanned and there was multiple mating of various domesticated breeds. However, in general, village chickens are small in terms of body size with different colors of plumage, and dual-purpose type (meat and egg), with varied physical characteristics and body conformation. Moreover, village chickens have a market weight of 1–1.5 kg, which can be reached in 4 to 5 months [18]. It is widely accepted that the meat and egg of village chickens have a better taste and stronger flavor compared to commercial broiler chickens, and they have therapeutic values. Therefore, the price of village chicken is higher than their commercial chicken counterparts, colored broiler breeds. Besides, as claimed by DVS, the number of farms providing village chicken all over Malaysia is 1015 farms; however, this number of farms is mixed with colored broiler chickens and there are no standard protocols or methods to differentiate between village chicken and colored broilers. Therefore, the DVS suspects that some of these farms are supplying colored broilers, while they claim they have village chicken. DVS mentioned that the lack of information regarding these two breeds can affect the pricing of village chicken since the colored broiler has a lower price compared to village chicken. Thus, lack of decisive strategy to differentiate between village chicken and colored broilers in Malaysia as well as the lower price of colored broiler compared to village chickens can lead to food fraud, in which some farms and market intermediaries provide underaged colored broiler as a village chicken to make a profit. Hence, in this study, two types of village chicken were investigated, including naturally mated village chicken (Ayam Kampung) (NMVC) and selective mated village chicken, which is named Akar Putra (APVC), to be compared with two underaged colored broiler breeds, including colored broiler-Hubbard (UCBH) and colored Broiler-Sasso (UCBS) for the purpose of village chicken authentication based on the phenotypic characteristics before and after slaughter. Since the sex effect was not significant for most variables in the first phase, in the second phase, only female chickens were considered for all four breeds.

There are different types of selective cross-breed village chicken in Malaysia [46]; however, in this study, the characteristics of Akar Putra, a new breed of village chicken grown by UPM, was investigated, which is claimed to be superior to the Ayam Kampung in that it can lay 200 eggs or 4 times as many as its free-range cousins. Besides, Akar Putra is bigger with more meat and longer legs, while taking a shorter time to reach maturity (13 weeks/1.2–1.4 kg) [50].

In the first phase, it was indicated that chicken breed is one of the major factors affecting phenotypic characteristics of chicken before and after slaughter. Similarly, the results of the second phase investigation showed that breed had a significant effect (*p* < 0.05) on measured phenotypes, except for dressing percentage and breast muscle weight. In other words, there were narrow variations in phenotypic characteristics among the second phase chicken breeds (NMVC, APVC, UCBH, UCBS), which approved the possible similarities in phenotypic characteristics of village chicken and underaged colored broiler breeds. The mean value ± standard deviation for nine variables is shown in Table 5.

#### 3.4.1. Body Weight and Linear Body Measurements

Regarding body size traits, two village chicken breeds were significantly different in terms of body length and shank length, while they were grouped together according to wingspan and chest circumference. Moreover, except for the shank length, which village chicken breeds had significantly (*p* < 0.05) longer shank compared to colored broiler breeds (Hubbard and Sasso), there was a minimum variation among these two breed groups based on other body size traits. Overall, NMVC had the longest body, shank, and wingspan compared to other chicken breeds. Even though chest circumference in APVC had a higher value and was significantly different from UCBS, there was no significant difference among chest circumference value in NMVC, APVC, and UCBH. It was obvious that both village chicken breeds (NMVC, APVC) had longer shank length compared to colored broilers, in which shank length is regarded as a good indicator of skeletal development and can be associated with chicken active walking potential to cover long distances in search of feed [51] (Table 5).

#### 3.4.2. Carcass Characteristics

Based on the results, UCBS had significantly higher breast muscle weight than its counterpart UCBH. Besides, there were no significant differences between the two village chickens (NMVC and APVC) and between the village chicken group and colored broiler group in terms of their breast muscle weight (Table 5). Therefore, breast muscle weight was not a suitable discriminator factor to differentiate village chicken breeds from colored broilers, even though colored broilers had the heavier breast muscle weight compared to the village chicken population.

Abdominal fat weight (AbFW) was significantly varied between village chicken and colored broiler breeds. As expected, both NMVC and APVC (5.38 and 13.34 g, respectively) had dominant lower AbFW compared with UCBH (23.58 g) and UCBS (29.91 g). Likewise, UCBS showed the greatest AbFW, being almost twice as large as APVC and five times greater than in NMVC, followed by UCBH (Table 5). Therefore, in this study, AbFW was one of the parameters that can significantly discriminate village chicken from underaged colored broiler breeds. Similarly, the study by Lokman et al. [6] indicated that indigenous Malaysian chickens were between Red jungle fowl and commercial broiler in terms of their fat content. Furthermore, our results approved the results of Muller et al., which revealed that broiler (Sasso 51) had a significantly higher abdominal fat proportion compared to the traditional dual-purpose breeds [27].

Although there was a negligible variation in live and carcass weight among NMVC, APVC, UCBS, and UCBH, there were no significant differences among the dressing percentage of all 4 breeds (Table 5). Thus, it can be concluded that all four chicken breeds had almost the same carcass yield after slaughter. However, NMVC and UCBS had a higher DP (67.58% and 66.42%, respectively) compared to other breeds. It should be noted that the mean value for breast weight in UCBS was nearer to the value in village chickens compared to UCBH. Accordingly, it leads to the conclusion that two village chickens and UCBS may share some important traits, including carcass yield and breast weight after slaughter, which can facilitate chicken adulteration for these breeds in the market.

Overall, in this study, NMVC had the highest mean value for body length, wingspan, shank length, and dressing percentage compared to other breeds, while the lowest mean value, which is for abdominal fat, belonged to NMVC (Table 5). Regarding APVC, there was no significant variation in phenotypic characteristics of this breed and NMVC, except for body and shank length. The findings in this study are similar to findings by Rofii et al. [14] who compared the physical characteristics of different Indonesian native chickens and showed that the body length was significantly different among populations. Subsequently, these two village chicken breeds (NMVC, APVC) can be significantly discriminated from underaged colored broiler chickens based on the two characteristics, including shank length and abdominal fat weight. However, other traits cannot be a powerful discriminating factor to differentiate village chicken and underaged colored broiler breeds. Thus, this study revealed that phenotypic characteristics based on the variables cannot be a reliable strategy to differentiate Malaysian village chickens from underaged colored broiler breeds.

#### 3.4.3. Intramuscular Fat Content Analysis

The mean value ± standard deviation of fat content in the breast muscle of female village chicken and underaged colored broiler breeds are shown in Table 6. Based on the measured values, the breed can be considered a factor with an effect on IMFP. However, in this study, UCBS was significantly differentiated with the highest IMFP from other breeds. On the contrary, the NMVC had the lowest mean value for IMFP and there were no significant differences among IMFP of NMVC, APVC, and UCBH.

According to Table 5 and Table 6, the underaged color broiler breeds had higher IMFP and AbFW content (high-fat content) compared to the village chicken breeds (low-fat content), in which the chicken fat content can be the discriminating factor for differentiating village chicken populations.

### 3.5. Principal Component Analysis

#### 3.5.1. First Phase

Principal component analysis (PCA) enables us to obtain a comprehensive idea of the relationship between different variables [52,53]. In this section, PCA was performed to discriminate groups belonging to different breeds based on the nine phenotypic variables and four selected chicken breeds. In fact, PCA was applied to facilitate the visualization of underlying data structure [33,54].

It has been proven in the previous section that different phenotypic characteristics can be used as the discriminating factors to differentiate different chicken breeds available in this study. Similarly, the results from score and loading plots showed the significant contribution of different variables in clustering chicken breeds in both phases.

**Figure 2:**
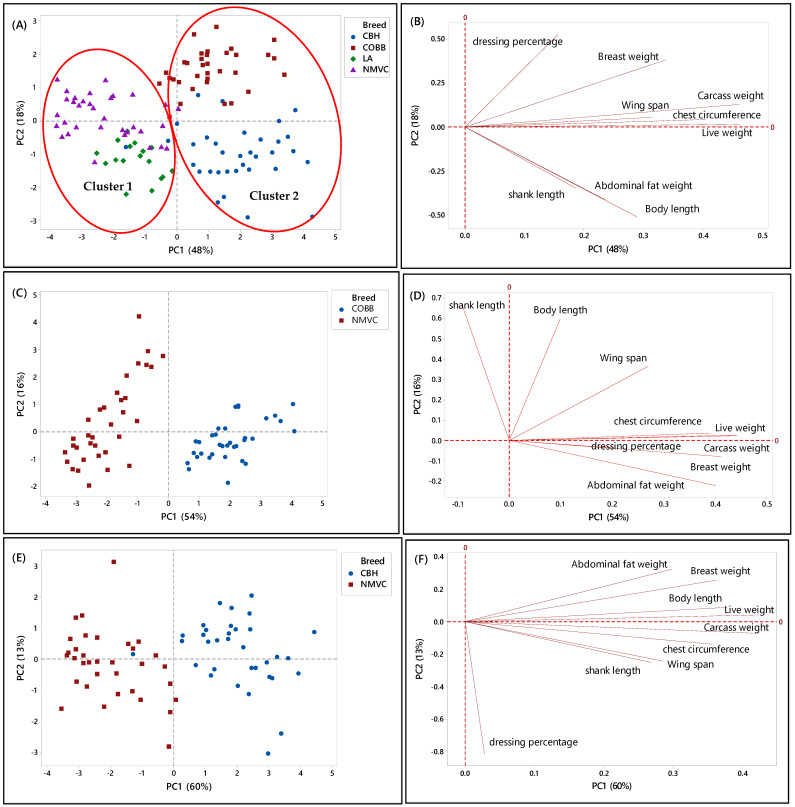

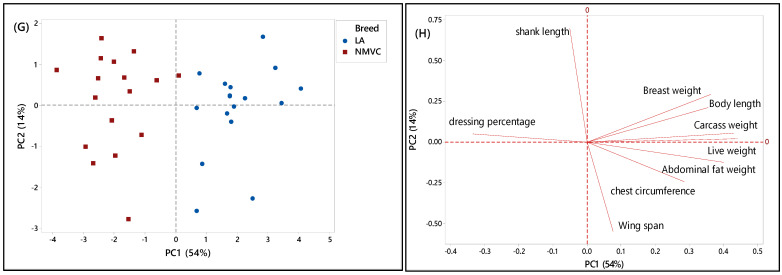
PCA score and loading plots showing the clustering pattern of different chicken breeds based on the nine phenotypic variables. (**A**,**B**) Score and loading plots of all breeds studied in the first phase, (**C**,**D**) naturally mated village chicken (Ayam Kampung) (NMVC) and Cobb (COBB), (**E**,**F**) naturally mated village chicken (Ayam Kampung) (NMVC) and Hubbard (CBH), and (**G**,**H**) naturally mated village chicken (Ayam Kampung) (NMVC) and layer-DeKalb (LA).

Likewise, Figure 2A–H, showed the relationship between the phenotypic characteristics and chicken breeds in the first phase. Accordingly, in Figure 2A, two principal components (PC) with eigenvalues of more than one were extracted to determine the best parameters that could discriminate chicken breeds, in which the PC1 and PC2 accounted for 48% and 18% of the total variation in the data, respectively. From this Figure, it can be observed that each chicken type (slow- and fast-growing) had specific clustering trends (95% confidence interval). Likewise, there were two clusters, one and two, which can be distinguished by virtue of the signs of the PC1 scores, in which chicken breeds in cluster 1 with a negative score of PC1 were slow-growing chickens, including naturally mated village chicken (Ayam Kampung) and DeKalb, were separated and discriminated from cluster 2 on a positive score of PC2, which were commercial broiler (Cobb) and colored broiler (Hubbard) chicken breeds. This clustering trend indicated that village chicken had significantly different phenotypic characteristics compared to broiler and colored broiler chicken breeds. Therefore, the phenotypic characteristics can be used as the potential criteria for the classification of chicken breeds. Moreover, according to the PC2, the commercial fast-growing lines, Cobb and Hubbard, can be separated on the positive and negative positions of PC2, respectively.

Alternatively, a loading plot had been taken into consideration to gain knowledge about variables and their correlation. Based on Figure 2B, variables including live weight, carcass weight, wingspan, and chest circumference, which were positively correlated together, had a large positive loading on PC1. Therefore, according to Table 2 and Figure 2B, these variables significantly differentiated commercial broilers (Cobb, Hubbard) from DeKalb and village chicken. Thus, these variables can be considered discriminating factors to separate fast-growing chicken breeds from slow-growing chicken breeds.

Moreover, Cobb and Hubbard were separated based on the PC2 (18 %), in which this close separation was contributed by significant differences in body length, shank length, and abdominal fat weight values with high negative loading on PC2. Hubbard population, which was located on the negative side of PC2, had a high value for body length, shank length, and abdominal fat weight. Therefore, these parameters can differentiate Hubbard from Cobb chickens. Besides, PC2 had a significant positive association with dressing percentage and breast muscle weight traits, and Cobb chicken located on a positive score of PC2; therefore, Cobb had a high value for these parameters compared to Hubbard.

To further understand the discriminating factors differentiating village chicken from other breeds, the score and loading plots separating village chicken from other breeds were shown in Figure 2C–H. As can be seen in Figure 2C,E, village chicken was significantly discriminated from Cobb and Hubbard based on the PC1, which accounted for 54% and 60% of the variance, respectively. Figure 2D indicated that village chicken had negative PC1 and was discriminated from Cobb chickens, on the positive side of PC1, by means of shank length trait. Based on Table 2, village chicken had a longer shank compared to the Cobb. Moreover, live weight, carcass weight, and chest circumference were positively correlated together, and along with breast weight and abdominal fat weight, located on a positive score of PC1, were the major discriminating factors that differentiate village chicken from Cobb, in which Cobb on the positive side of PC1 had a higher value for all mentioned variables compared to village chicken. Based on Figure 2C, the most significant discriminating factors separating village chicken from Hubbard in virtue of PC1 were live weight, carcass weight, breast weight, body length, chest circumference, and abdominal fat weight, which all had higher value in Hubbard compared to village chickens.

As mentioned earlier, in this study, only female layers have been considered for the analysis. Therefore, Figure 2G showed dominant differentiation of female village chicken from female DeKalb in virtue of PC1 (54%), which was positive for DeKalb cluster and negative for village chicken population. Figure 2H showed that the variables including live weight, carcass weight, body length, and abdominal fat weight, which had the highest value on a positive score of PC1 in DeKalb, can be considered as the discriminating factors to differentiate village chicken from DeKalb. Whereas, dressing percentage and shank length, which were negatively correlated with PC1, had a higher value in village chicken compared to DeKalb.

Overall, the results showed that there were notable differences in phenotypic characteristics of different chicken breeds in this study that enable us to differentiate village chickens as a precious breed from other chicken breeds. However, although the village chicken population can be differentiated from commercial broiler (Cobb and Hubbard) based on the significant discriminating factors, including live weight, carcass weight, breast weight, chest circumference, and abdominal fat weight, they were grouped with DeKalb in cluster 1 on the negative side of PC1. This grouping pattern can be explained by the growth rate of DeKalb, which were considered as a slow-growing breed and had almost the same phenotypic characteristics, such as breast weight, chest circumference, and wingspan, as village chicken. Moreover, the intra-population variation can be seen in some score plots, such as Figure 2G, which showed the variation in phenotypic characteristics of individuals in the village chicken and DeKalb population.

#### 3.5.2. Second Phase

In the second phase, PCA was applied to a matrix of nine analytical variables for four chicken breeds to visualize the discriminating and grouping pattern of two Malaysian village chickens (NMVC, APVC) compared to underaged colored broiler breeds (UCBH, UCBS). Therefore, this chicken breed clustering can facilitate us in discriminating village chickens from their fraudulent counterparts, underaged colored broiler breeds (UCBH, UCBS), which has been the current issue of food safety in Malaysia.

**Figure 3:**
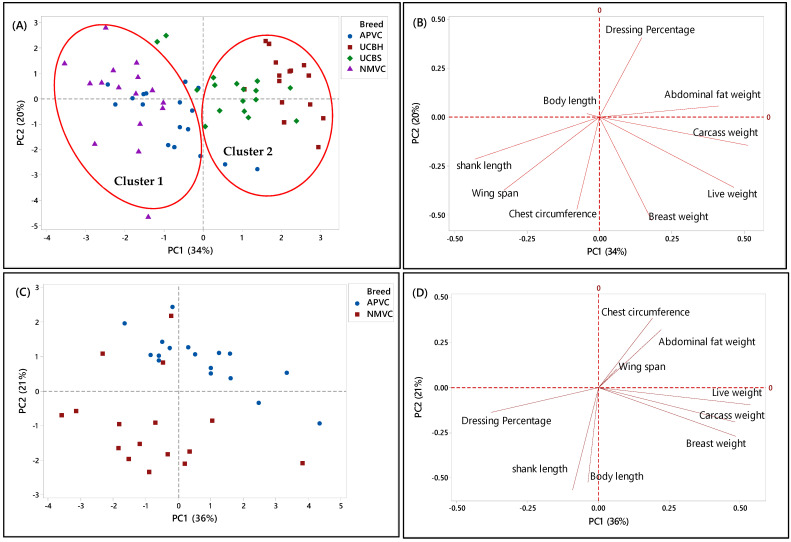

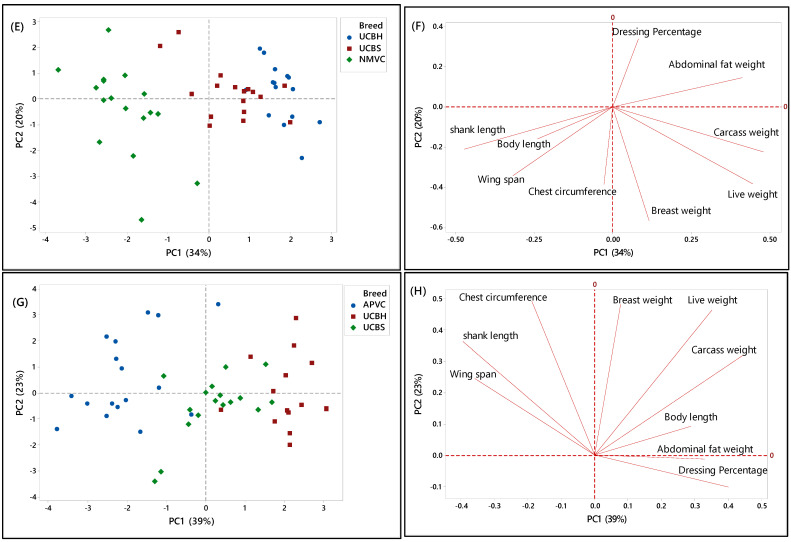
PCA score and loading plots showing the clustering pattern of different chicken breeds based on the nine phenotypic characteristics. (**A**,**B**) Score and loading plots of all breeds studied in the second phase, (**C**,**D**) naturally and selective mated village chickens (NMVC, APVC), (**E**,**F**) naturally mated village chicken (Ayam Kampung) (NMVC) and underaged colored broilers (UCBH, UCBS), and (**G**,**H**) selective mated village chicken-Akar Putra (APVC) and underaged colored broilers (UCBH, UCBS).

A complicated link between the breed varieties and their phenotypic characteristics was observed based on the PCA results in both score and loading plots in Figure 3A–H. It has been indicated that several characteristics can be used as discriminating factors to differentiate village chicken from underaged colored broiler breeds. Figure 3A showed that both village chicken populations (APVC and NMVC) were dominantly grouped (cluster 1) and significantly separated from UCBS and UCBH in cluster 2 based on the PC1. This revealed that phenotypic characteristics can be effective in differentiating the village chicken from colored broiler breeds. Accordingly, village chickens had higher values for shank length, wingspan, and chest circumference, whereas loading on abdominal fat weight, breast weight, and live and carcass weight dominated the underaged colored broiler group (Figure 3B). However, although two underaged colored broiler breeds were dominantly positioned on the positive side of the PC1 and separated from village chickens, there were partial overlaps of UCBS observations with those of APVC in the village chicken cluster. This pattern showed that UCBS may share some phenotypic characteristics with two village chicken groups, specifically with the selective breed (APVC), which may make it difficult to authenticate village chickens from underaged colored broiler. Similarly, the data in Table 5 showed that APVC and UCBS had approximately the same range value for various traits, including body length, wingspan, live weight, carcass weight, breast weight, and dressing percentage.

Another PCA was performed for further identification of significant discriminating factors separating village chicken populations from underaged colored broiler breeds. The score and loading plots discriminating village chickens were shown in Figure 3C–H. Likewise, Figure 3E,G showed that PC1 is responsible for separating village chicken groups from underaged colored broilers based on their phenotypic characteristics. In both Figures, PC1 scores were negative for village chickens and positive for underaged color broilers. According to Figure 3E,F, the most significant discriminating factors differentiating village chicken (NMVC) from colored broiler breeds were shank length and abdominal fat weight. Similarly, APVC can be significantly separated from UCBH and UCBS based on shank length and abdominal fat weight traits (Figure 3G,H). However, as it is obvious in Figure 3G, there were overlaps between observations for APVC and UCBS, which pointed out that these two breeds share characteristics such as body length, wingspan, carcass weight, dressing percentage, and breast weight (Table 2).

Overall, there were narrow differences among phenotypic characteristics of village chicken and underaged color broiler, which makes the discrimination of these two breeds more complicated. The only significant factors that can be used for differentiation were chest circumference, shank length, which had a higher value in village chicken groups, and abdominal fat content, with higher value in the colored broiler breeds. However, the most important factors, which enabled after slaughter’s discrimination, such as breast muscle weight and dressing percentage, were approximately in the same range in both chicken breed groups.

Figure 3C,D illustrated the separation of two village chicken groups and the discriminating factors responsible for this separation. PC2 (21%) was responsible for separating NMVC from APVC. Based on Figure 3D, body length and shank length, which were positively correlated, were significant discrimination factors, while other phenotypes almost had the same value in both breeds. Besides, in Figure 3C, the intra-population variation can be observed for both village chicken (NMVC, APVC), which revealed the higher diversity in phenotypic characteristics of individuals in both village chicken populations [55].

## 4. Conclusions

Since the village chicken industry has not been well-characterized in Malaysia, this study aimed to phenotypically characterize and differentiate village chicken breeds from their counterparts, including commercial broilers, layer, and underaged colored broilers. Appropriate characterization of Malaysian village chicken breeds will promote their commercialization while improving their productivity through aiding in the genetic selection of higher-performing chickens. The current study revealed clear breed clustering, which leads to the authenticity of chickens based on their phenotypic characteristics before and after slaughter.

The results of the first phase indicated that chicken breed had a significant effect (*p* < 0.05) on all phenotypic characteristics under investigation. Thus, different economically important phenotypes can be highly influenced by breeds and the genetic background of the chickens. The most remarkable differentiating factors, such as breast muscle weight, chest circumference, carcass weight, abdominal fat, and Intramuscular fat weight, can be significant criteria for grouping and differentiation of village chicken populations from commercial broiler chicken populations, whereas sex just significantly affected body length, shank length, chest circumference, and live and carcass weight. In the second phase, the Malaysian slow-growing village chicken populations differentiated from underaged colored broiler breeds based on the different phenotypic characteristics, except for chest circumference, breast muscle weight, and dressing percentage. Therefore, since Malaysian village chickens had the same carcass yield and breast muscle weight as two underaged colored broilers, they can be easily substituted for colored broiler after slaughter to deceive the consumers. The PCA results showed that chicken breed clustering will facilitate us in discriminating village chickens from their fraudulent counterparts, underaged colored broiler breeds in the second phase and high-performance chicken breeds (broiler and colored broiler breeds) in the first phase, based on their phenotypic traits. Outcomes of this study can benefit the Department of Veterinary section (DVS), farmers, and chicken consumers to differentiate Malaysian village chicken as a precious breed from other chicken breeds available in the market. However, since no attempt has ever been made to characterize local chicken populations phenotypically and genetically in Malaysia, there is a need for a comprehensive study to investigate all village chicken breeds available in all 1015 farms that claimed to supply village chicken. Generally, chicken phenotypic characterization is the first step to promote their usage, conservation of local chicken genetic diversity, and preservation of further adulteration.

## Figures and Tables

**Table 1:** Nutrient composition of feed.

Item	Proportion (%)	
	Starter	Grower
Crude protein	21	19
Crude fiber	5	5
Crude fat	4	5
Moisture	13	13
Ash	8	8
Calcium	0.8	0.8
Phosphorous	0.4	0.4

**Table 2:** Effect of breed and sex on the live weight, body size, and carcass characteristics of four chicken breeds in Malaysia.

Phenotypes	Breed (B)				Sex (S)		*p*-Value		
	Broiler-Cobb	Colored Broiler-Hubbard	Layer-DeKalb	Village Chicken	Male	Female	B	S	B × S
BL (cm)	34.17 ± 1.88 ^c^	42.47 ± 3.83 ^a^	37.35 ± 2.39 ^b^	34.10 ± 3.46 ^c^	38.58 ± 5.37 ^A^	35.24 ± 4.10 ^B^	0.00	0.00	0.006
WS (cm)	45.08 ± 1.86 ^a^	46.82 ± 5.23 ^a^	40.59 ± 5.20 ^b^	40.62 ± 7.14 ^b^	45.17 ± 5.91 ^A^	43.17 ± 5.56 ^A^	0.00	0.052	0.276
SL (cm)	9.38 ± 0.81 ^c^	11.11 ± 0.94 ^a^	9.17 ± 0.80 ^c^	10.26 ± 1.16 ^b^	10.75 ± 1.14 ^A^	9.75 ± 1.06 ^B^	0.00	0.00	0.023
CC (cm)	37.94 ± 3.02 ^a^	38.76 ± 5.34 ^a^	33.29 ± 3.77 ^b^	30.5 ± 3.92 ^b^	38 ± 5.66 ^A^	33.46 ± 4.53 ^B^	0.00	0.00	0.037
LW (g)	2609.7 ± 390.6 ^a^	2710.8 ± 457.9 ^a^	1819.3 ± 147 ^b^	1272.5 ± 292.7 ^c^	2476.1 ± 761 ^A^	1919.2 ± 659.4 ^B^	0.00	0.00	0.218
CW (g)	1872.2 ± 286.6 ^a^	1861 ± 347.4 ^a^	1105.4 ± 102 ^b^	872.5 ± 211.5 ^c^	1741.1 ± 549.8 ^A^	1329.4 ± 472 ^B^	0.00	0.00	0.123
DP (%)	71.81 ± 3.78 ^a^	68.53 ± 3.63 ^b^	60.76 ± 2.92 ^c^	68.60 ± 4.23 ^b^	70.23 ± 4.26 ^A^	69.06 ± 3.98 ^A^	0.00	0.123	0.108
BW (g)	489.7 ± 117.1 ^a^	359.97 ± 57.45 ^b^	248.82 ± 42.2 ^c^	215.91 ± 48.22 ^c^	370.7 ± 147.7 ^A^	339.71 ± 126.4 ^A^	0.00	0.051	0.333
AbFW (g)	29.28 ± 7.55 ^b^	72.57 ± 44.41 ^a^	58.52 ± 27.18 ^a^	0.335 ± 0.87 ^c^	29.33 ± 30.14 ^A^	38.80 ± 46.72 ^A^	0.00	0.063	0.144

Data are mean ± standard deviation; ^a,b,c,A,B^ Means within a row with different superscripts differ significantly (*p* < 0.05); BL = Body length, WS = Wing span, SL = Shank length, CC = Chest circumference, LW = Live weight, CW = Carcass weight, DP = Dressing percentage, BW = Breast weight, AbFW = Abdominal fat weight.

**Table 3:** Effect of breed and sex on intramuscular fat percentage of breast muscle in four chicken breeds in Malaysia.

Phenotype	Breed (B)				Sex (S)		*p*-Value		
	Broiler-Cobb	Colored Broiler-Hubbard	Layer-DeKalb	Village Chicken	Male	Female	B	S	B × S
IMFP	2.852 ± 0.96 ^b^	2.443 ± 0.72 ^b^	3.617 ± 0.80 ^a^	1.240 ± 0.39 ^c^	2.04 + 0.99 ^A^	2.31 ± 1.0 ^A^	0.00	0.156	0.09

Data are mean ± standard deviation; ^a,b,c,A^ Means within a row with different superscripts differ significantly (*p* < 0.05). IMFP = Intramuscular fat percentage.

**Table 4:** Comparison of IMFP in different sexes in three chicken populations.

Sex	Broiler-Cobb	Color Broiler-Hubbard	Village Chicken
Male	2.921 ± 1.09 ^a,A^	2.030 ± 0.33 ^a,B^	1.189 ± 0.43 ^a,C^
Female	2.783 ± 0.87 ^a,A^	2.856 ± 0.78 ^b,A^	1.292 ± 0.37 ^a,B^

Data are mean ± standard deviation. Means within a row ^A,B,C^ and column ^a,b^ with different superscripts differs significantly (*p* < 0.05). IMFP = Intramuscular fat percentage.

**Table 5:** Effect of breed on live weight, body size, and carcass characteristics of Malaysian village chickens and two underaged colored broiler breeds.

Phenotypes		Breed (B)			*p*-Value
	Village Chicken-Ayam Kampung	Village Chicken-Akar Putra	Colored Broiler-Hubbard	Colored Broiler-Sasso	Breed
BL (cm)	32.147 ± 2.63 ^a^	28.706 ± 2.31 ^c^	30.941 ± 2.19 ^ab^	29.412 ± 1.27 ^bc^	0.00
WS (cm)	38.47 ± 5.22 ^a^	37.588 ± 2.57 ^ab^	32.176 ± 1.87 ^c^	35.176 ± 1.74 ^b^	0.00
SL (cm)	9.441 ± 0.68 a	7.353 ± 0.72 b	5.294 ± 0.73 c	5.852 ± 0.23 c	0.00
CC (cm)	28.971 ± 4.04 ^ab^	30.176 ± 2.43 ^a^	28.176 ± 1.87 ^ab^	27.529 ± 1.80 ^b^	0.035
LW (g)	1053.9 ± 177.5 ^b^	1154 ± 133.2 ^b^	1333.6 ± 87 ^a^	1155.9 ± 102.9 ^b^	0.00
CW (g)	706.5 ± 92 ^b^	724.3 ± 85.8 ^b^	877.5 ± 165.3 ^a^	767.3 ± 73.6 ^b^	0.00
DP (%)	67.58 ± 4.90 ^a^	62.76 ± 1.80 ^a^	65.76 ± 11.48 ^a^	66.42 ± 3.42 ^a^	0.181
BW (g)	196.25 ± 29.36 ^ab^	196.12 ± 25.20 ^ab^	175.8 ± 46.6 ^b^	205.33 ± 23.95 ^a^	0.067
AbFW (g)	5.38 ± 10.35 ^b^	13.34 ± 9.01 ^b^	23.58 ± 9.53 ^a^	29.91 ± 12.86 ^a^	0.00

Data are mean ± standard deviation. ^a,b,c^ Means within a row with different superscripts differ significantly (*p* < 0.05). BL = Body length, WS = Wing span, SL = Shank length, CC = Chest circumference, LW = Live weight, CW = Carcass weight, DP = Dressing percentage, BW = Breast weight, AbFW = Abdominal fat weight.

**Table 6:** Effect of breed on intramuscular fat content of breast muscle in four chicken breeds.

Phenotype	Breed (B)				*p*-Value
	Underaged Colored Broiler-Sasso	Underaged Colored Broiler-Hubbard	Village Chicken-Akar Putra	Village Chicken-Ayam Kampung	Breed
IMFP	3.158 ± 0.9 ^a^	1.846 ± 0.3 ^b^	1.482 ± 0.2 ^b^	1.292 ± 0.3 ^b^	0.00

Data are mean ± standard deviation. ^a,b^ Means within a row with different superscripts differ significantly (*p* < 0.05).

## Data Availability

Not applicable.
